# Annotations

**Published:** 1890-12-13

**Authors:** 


### Annotations.

**Dr. Sarah E. Post.**

Dr. Sarah Post is the editor or editress of *The Nightingale*, a a small periodical published in New York in the interests of good nursing. Dr. Sarah Post sets forth in *The Nightingale*, of November 15th, that there is "something wrong in England," and the basis of her logical pyramid is that The Hospital has advanced the opinion that lady doctors are "in actual practice less thorough, less courageous, and less capable than men." Dr. Sarah Post does not venture to traverse these opinions in any way—she leaves them severely, and perhaps wisely alone. But she does not leave The Hospital alone for all that. We had ventured to affirm that motherliness is one of the desirable endowments of a doctor. Here was the lady editor's opportunity, and she was not slow to seize upon it. Feeling that the main position occupied by The Hospital was too strong for her, she set about executing a slashing attack on the remotest outpost. That was journalistically clever, and furnished an excellent opportunity for a brilliant display of lady medical polemics. But it was not business, and it certainly was not war, for, as we stated at the outset, our main position was not so much as attempted to be carried. But we would like Dr. Sarah Post, and all other lady medicals to regard us as friendly and not as adverse critics. Our article was written with the object of pricking the sides of the good intent of the lady doctors. If we return to the charge, as we do, it is only that the ladies practising medicine may see themselves as others see them, and may at once take steps to raise their work to the level of the best medical and surgical practice of the times. We offer a courteous challenge to Dr. Sarah Post; our position is, that in medicine the best lady practitioners are much inferior to the best physicians of the other sex; and that in surgery they are simply nowhere. This is a fair challenge, and we hope it will be accepted by Dr. Sarah Post, or one of her collaborateurs; and that in the pages of an early issue of *The Nightingale* we may have notes of sound logic and of sturdy literary battle, without any attempts to ride off on side issues. If our challenge is taken up, we, on our part, promise to maintain our thesis in a true and knightly spirit. In the meantime, we show our desire to acknowledge the equality of the sex with our own by our challenge, and so "there lies our glove."

**An Honorary and an Honourable Secretary.**

For about twenty years past Mr. W. H. Wheeler has been the honorary, and the only, secretary of the Boston Hospital. Mr. Wheeler's work in connection with that institution has been so considerable and so valuable that it not only calls for grateful record, but offers an example which may be followed with great public advantage in many other towns beside Boston. Twenty years ago Boston had no hospital at all, and what a deprivation that was to the poor, who thus lacked skilful treatment, and to the better classes, who had no scientific medical centre for the instruction and stimulation of their doctors, only those know who are familiar with hospital affairs. The first attempt to supply Boston with hospital accommodation was made by Mr. Wheeler and a few friends. All that could be accomplished at that time was the securing of one or two small houses on the bank of the river, and the conversion of them into a cottage hospital. How that hospital was equipped we need not ask, but it is very rare indeed that "converted cottages" make efficient hospitals. At the present time, instead of the ill-adapted and poorly-equipped cottages on the river bank, there is at the southern end of the town a noble pile of buildings, which adorn the street, and which confer upon the poor and, indirectly upon the rich, the untold benefits of modern medical science. To Mr. Wheeler first and chiefly does the honour of achieving this great work belong. The occasion of our remarks is that Mr. Wheeler, through failing health, is compelled to relinquish his honorary and honourable duties. Although such relinquishment is almost a daily occurrence with valued public servants, yet it is none the less an occasion of loss and sadness. All the other officers and friends of the institution sorrow at the loss of Mr. Wheeler's services, and at his failing health which compels that loss. The public of Boston, whose trusted almoner he has been, share in the sorrow. Those feelings are honourable both to Boston and to Mr. Wheeler, and are the natural and just reward for his work. Many hospitals would be gainers, and the work of charity would be much enlarged, if gentlemen of means and position in other towns would consider the example of men like Mr. Wheeler, and make some effort to "go and do likewise."

**Dr. Rentoul's Crusade against Midwives.**

There is a measure before Parliament which has for its object the registration of midwives. The reason why certain public-spirited persons persons wish to have midwives registered is that they desire to secure for those poor women who cannot afford to pay the fees of doctors, the attendance of properly skilled persons of their own sex in confinement. This seems to us, as it does to most people, a just and humane object. It is an object which those who know most about the question entirely approve. The medical men of the United Kingdom, who are better qualified than any other class to pass judgment on the subject, give it an almost universal approbation. But there are exceptions to this rule, and Dr. Rentoul, of Liverpool, is one of them. We have nothing to say against Dr. Rentoul in his private capacity as a medical practitioner, but there is very much to be said against the *rôle* he has assumed as leader of a scattered battalion of malcontents against a measure which is calculated to be of great public utility. We can very well understand that Dr. Rentoul and a very limited number of general practitioners of the poorest sort will have an objection to the proposed registration of midwives, on the ground that it may prevent, here and there, a few small midwifery fees from coming into their pockets. And if Dr. Rentoul, as the champion of this handful of opponents, would honestly and squarely say that his opposition has its ground in the fee question, we could at least respect his candour though we could not approve his judgment. But instead of this he besieges the offices of medical newspapers and the breakfast tables of private practitioners with reams of octavo, crammed with all sorts of pertinent and impertinent objections to midwives registration, gathered from the ends of the earth. To answer these objections in detail would take hours of time and volumes of paper, and would be as needless and useless a task as that of Sisyphus, who constantly rolled a huge stone to the top of a hill with no other result but that of seeing it roll down again faster than he had rolled it up. The enormous majority of medical practitioners will, we are persuaded, repudiate Dr. Rentoul and his reams of reasons as heartily as we do. They do not want to attend confinements, for which inadequate fees are paid—they cannot afford to do so. Such confinements drag them out of bed at night, and do them ten times more harm than the miserable fees are worth. And yet they do not feel happy in refusing to attend the very poor in confinements when they are asked. As a matter of fact, the majority of practitioners, as has been shown by the action of several branches of the British Medical Association, will be relieved both in mind and body when Parliament, by its Midwives' Registration Bill, shall have secured for the women of the poorer classes something like skilled treatment at the time of Nature's distress and anguish. In the meantime, Dr. Rentoul will do well to cease from his kite-flying. He gives the impression that he is much more anxious to pose as the leader of the general practitionors of medicine than to serve the interests of the poor women about whom he strives to appear so profoundly concerned.

